# Second victim experiences and moral injury as predictors of hospitalist burnout before and during the COVID-19 pandemic

**DOI:** 10.1371/journal.pone.0275494

**Published:** 2022-10-04

**Authors:** Tejasri Chandrabhatla, Henok Asgedom, Zehra P. Gaudiano, Leyla de Avila, Kenneth L. Roach, Chapy Venkatesan, Ali A. Weinstein, Zobair M. Younossi

**Affiliations:** 1 Department of Medicine, Inova Fairfax Hospital, Fairfax, VA, United States of America; 2 Department of Global and Community Health, College of Health and Human Services, George Mason University, Fairfax, VA, United States of America; 3 Betty and Guy Beatty Center for Integrated Research, Inova Health System, Falls Church, VA, United States of America; 4 Center for the Advancement of Well-Being, George Mason University, Fairfax, VA, United States of America; Hackensack Meridian Health, UNITED STATES

## Abstract

**Background:**

The increasing number of physicians leaving practice, especially hospitalists, has been well-documented. The most commonly examined factor associated with this exodus has been burnout. The COVID-19 pandemic has put a unique and unprecedented stress on hospitalists who have been at the front lines of patient care. Therefore, the investigation of burnout and its related factors in hospitalists is essential to preventing future physician shortages.

**Objective:**

This study examined the relationship between burnout, second victim, and moral injury experiences before and during the COVID-19 pandemic among hospitalists.

**Methods:**

Two anonymous cross-sectional surveys of hospitalists from a community hospital in the metropolitan Washington, DC area were conducted. One was conducted pre-COVID-19 (September-November 2019) and one was conducted during COVID-19 (July-August 2020). The surveys were sent to all full-time hospitalists via an online survey platform. A variety of areas were assessed including demographic (e.g., age, gender), work information (e.g., hours per week, years of experience), burnout, second victim experiences, well-being, and moral injury.

**Results:**

Burnout rates among providers during these two time periods were similar. Second victim experiences remained prevalent in those who experienced burnout both pre and during COVID-19, but interestingly the prevalence increased in those without burnout during COVID-19. Moral injury was predictive of burnout during COVID-19.

**Conclusion:**

While there were some factors that predicted burnout that were similar both pre- and during-pandemic, moral injury was unique to predicting burnout during COVID-19. With burnout as a contributing factor to future physician shortages, it is imperative that predictive factors in a variety of different environments are well understood to prevent future shortages. Hospitalists may be an excellent barometer of these factors given their presence on the front line during the pandemic, and their experiences need to be further explored so that targeted interventions aimed at addressing those factors may be created.

## Introduction

The U.S. faces a significant physician shortage with recent projections quantifying the shortage as 122,000 physicians by 2032 [1–3]. Physician burnout is one potential contributing factor to this projected shortage of physicians [4,5]. Burnout is a condition in which an individual experiences depersonalization, emotional exhaustion, and feeling devoid of personal achievement in the workplace [6,7]. The onset of burnout has been tied to multiple negative outcomes for both physicians and their patients [8–11]. Burnout in physicians has been linked with an increase in risk for medical errors [10,11], higher physician turnover rates [12,13], and a substantial increase in suicidal ideation for physicians [14].

Burnout is one potential factor that contributes to physicians leaving practice [15–17], but other factors can also contribute to those decisions. For example, other factors that may contribute to physicians’ satisfaction with their profession are second victim experiences [18,19], moral injury[20,21], and overall well-being [22,23]. Second victim experiences occur when health care providers are involved in an unexpected negative outcome for a patient, which subsequently traumatizes the provider [24,25]. Moral injury describes the negative mental health impacts of events that cause a provider to feel they have violated a certain moral code [26,27]. Second victim experience and moral injury have typically been assessed in extreme settings like warzones and healthcare crises [28,29]. These two phenomena can be related but they are separate constructs. One can experience a second-victim experience without moral injury. For example, a medical error that harms a patient has been related to second victim experiences for health professionals [30]. However, since moral injury is defined by going against one’s values and moral beliefs, medical error would not necessarily be related to moral injury.

Different physician specialties may have different levels and predictors of burnout, however, not all specialties have been studied equally. One group of physicians that have previously been understudied are hospitalists. With the healthcare crisis of COVID-19, there has been a lasting concern for the pandemic’s effect on front-line workers and their mental health [31–33]. Hospitalists have been at the forefront of the response to the pandemic, and as mentioned above, have been relatively understudied with regards to burnout and other factors. There is little data available investigating the pre-COVID-19 state of these factors and even less data available on the potential changes in these factors in response to the pandemic. Therefore, the objective of this research is to investigate burnout, second victim experience, and moral injury in hospitalists before and during the COVID-19 pandemic.

## Methods

Anonymous surveys were sent to all full-time hospitalists at a community hospital in the Metropolitan Washington, DC area via an online survey platform at two different time points: (1) between September and November 2019, pre-COVID-19; and (2) between July and August 2020, during COVID-19. The research was approved by the hospital’s Institutional Review Board, including an online informed consent process. Respondents had to respond that they agreed to continue to complete the survey after being presented with the informed consent document. The responses to the survey had no identifying information and could not be associated with individual physicians.

### Survey

A variety of areas were assessed in the survey including demographic (e.g., age, gender), work information (e.g., hours worked per week, years of experience), burnout, second victim experiences, well-being, work well-being, moral injury, and perceived COVID-19-related risk (only assessed during COVID-19).

### Burnout

Burnout was assessed in two ways. First, the one-item Mini Z Burnout Survey was used to identify the presence of burnout [34]. This item asks: *“Overall*, *based on your definition of burnout*, *how would you rate your level of burnout*?*”* Responses are scored on a five-category ordinal scale, where 1 = *“I enjoy my work*. *I have no symptoms of burnout;”* 2 = *“Occasionally I am under stress*, *and I don’t always have as much energy as I once did*, *but I don’t feel burned out;”* 3 = *“I am definitely burning out and have one or more symptoms of burnout*, *such as physical and emotional exhaustion;”* 4 = “*The symptoms of burnout that I’m experiencing won’t go away*. *I think about frustration at work a lot;”* and 5 = *“I feel completely burned out and often wonder if I can go on*. *I am at the point where I may need some changes or may need to seek some sort of help*.*”* This item was dichotomized as ≤2 (no burnout) vs. ≥3 (having burnout) [34,35]. In addition, the Abbreviated Maslach Burnout Inventory was used [36]. There are 9 total items with 3 items per subscale: Emotional Exhaustion, Depersonalization, and Personal Accomplishment. This scale has previously been used for burnout evaluation in physicians [37].

### Second victim experiences

Second victim experiences were assessed with the Second Victim Experience and Support Tool. These were defined as “an unanticipated adverse patient event, medical error and/or a patient-related injury that traumatizes the provider” [38]. Individuals that endorsed the question: “In the past 12 months, were there any clinical events that caused personal problems, such as anxiety, depression, or concern about your ability to perform your job” were considered to have had a second victim experience [39].

### Well-being

Overall well-being was assessed with two instruments, the Flourishing Scale and the Satisfaction with Life Scale. The Flourishing Scale is a brief 8-item summary measure of self-received success in important areas such as relationships, self-esteem, purpose, and optimism [40]. The Satisfaction with Life Scale is a short 5-item instrument designed to measure global cognitive judgments of satisfaction with one’s life [41].

### Work well-being

Work well-being was assessed with the Work Well-Being Scale [42] and satisfaction with work-life balance was assessed by the item “My work schedule leaves me enough time for my personal/family life” (response options: strongly agree, agree, neural, disagree, and strongly disagree) [4,43]. Individuals who indicated “disagree” or “strongly disagree” were considered to be dissatisfied with their work-life balance [4,43].

### Moral injury

The Moral Injury Events Scale is a nine-item assessment of moral injury [44,45]. It was designed to be used with the armed forces. We adapted the two questions that specifically referred to the military in order to make it relevant to physicians. Specifically, in question 8, “fellow service members” was changed to “fellow medical professionals” and in question 9 “U.S. military” was changed to “medical profession”.

### Perceived COVID-19-related risk

A nine-item questionnaire that assessed perceived SARS-related risk, was adapted to assess COVID-19-related risk [46,47]. Simply, wherever “SARS” was mentioned in the survey, it was replaced with “COVID-19.” The items addressed during-outbreak perceptions of COVID-19-related threat (e.g., “I believed that my job was putting me at great risk”).

### Statistical analysis

Standard descriptive summary statistics were used to characterize the sample. Independent samples t-tests were used to compare pre-COVID-19 to during COVID-19 time points on sample characteristics, burnout, well-being, second victim experiences and moral injury. Independent samples t-tests were then used to compare those with and without burnout, pre-COVID-19 and during COVID-19 on the same self-report questionnaires, with additional t-tests being used for an item analysis of the Moral Events Injury Scale. All tests were two-tailed, with type 1 error level of 0.05. Analyses were performed with SPSS version 26 (IBM Corp., Armonk, NY).

## Results

Forty-four hospitalists (out of 54, response rate of 81%) completed the survey prior to the start of COVID-19 (between September and November 2019) and 37 hospitalists (out of 58, response rate of 64%) completed the survey during COVID-19 (between July and August 2020). Hospitalist characteristics are presented in Table 1. The majority of characteristics were not statistically significantly different comparing pre-COVID-19 to during COVID-19, with the exception of hours worked per week which decreased during COVID-19 (pre: 53.1±13.7 vs. during: 47.5±8.1, p = 0.035) and percentage of time spent in non-clinical work activities which decreased during COVID-19 (pre: 15.4%±17.1 vs during: 8.8%±11.7, p = 0.045).

**Table 1:** Characteristics of hospitalists.

		Pre-COVID-19 n = 44	During COVID-19 n = 37	p-value
Age	20–40	34 (75%)	27 (69%)	0.670
	41–50	8 (18%)	10 (26%)	
	51–60	3 (7%)	2 (5%)	
Gender (Female)		19 (42%)	21 (54%)	0.547
Relationship Status	Stable partner/married	34 (77%)	31 (79%)	0.759
	Divorced	2 (5%)	1 (3%)	
	Single	7 (16%)	7 (18%)	
Children (Yes)		25 (57%)	24 (62%)	0.663
Number of children		2.0 (0.8)	1.9 (0.8)	0.578
Years since residency		7.3 (5.1)	7.4 (5.3)	0.960
Years as a hospitalist		6.6 (4.4)	6.8 (4.8)	0.865
Hours worked per week		53.1 (13.7)	47.5 (8.1)	**0.035**
Percentage of work time spent in the following activities	Clinical work, non-teaching service	72.1 (27.3)	73.4 (31.0)	0.842
	Clinical work, teaching service	12.5 (19.4)	13.3 (23.7)	0.864
	Non-clinical work	15.4 (17.1)	8.8 (11.7)	**0.045**
Percentage of clinical time spent in the following activities	Rounding and discharging	51.2 (37.1)	53.0 (37.5)	0.833
	Admitting during the day	12.5 (18.4)	10.5 (11.6)	0.570
	Admitting during the night	19.9 (31.8)	20.0 (31.0)	0.990
	Emergency department triage	8.7 (17.9)	7.3 (11.8)	0.666
	Cross cover	5.5 (8.6)	3.4 (7.4)	0.246
	Other	2.2 (9.3)	1.8 (8.0)	0.813
My clinical workload exceeds a safe level	Never	2 (5%)	7 (18%)	0.084
	Once a year	5 (11%)	4 (11%)	
	Once a month	20 (45%)	19 (50%)	
	Once a week	9 (20%)	7 (18%)	
	Daily	8 (18%)	1 (3%)	

Data are presented as counts (percentages) or means (standard deviation).

p-value < 0.05 was considered statistically significant.

As seen in Table 2, there were no statistically significant differences in levels of distress, burnout, and well-being comparing hospitalists pre and during COVID-19. For both pre and during COVID-19 time points, 43% of the respondents reported burnout. Table 3 examines factors among those with and without burnout pre and during the pandemic. Pre-COVID-19, emotional exhaustion and depersonalization were statistically significantly different between those with and without burnout. These factors are considered part of the burnout phenomenon. In addition, second victim experiences, work-life balance, work well-being, and flourishing (overall well-being) were also statistically significantly different between burnout groups. During COVID-19, emotional exhaustion, depersonalization, and work well-being were statistically significantly different between those with and without burnout, as also seen pre-COVID-19. However, other factors were statistically significantly different between burnout groups during COVID-19 that were not different pre-COVID-19, which were satisfaction with life, moral injury, and perceived COVID-19-related risk (not assessed pre-COVID-19). Within the perceived COVID-19-related risk scale, the one question that was different between those with and without burnout was “I believe that my job was putting me at great risk for contracting COVID-19,” with 94% of those with burnout endorsing that question compared to 62% without burnout.

**Table 2:** Distress, burnout and well-being for hospitalists pre and during COVID-19.

	Pre COVID-19 n = 44	During COVID-19 n = 37	p-value
Emotional Exhaustion	8.7 (4.3)	8.5 (3.9)	0.899
Depersonalization	4.9 (4.4)	3.5 (3.5)	0.132
Personal Accomplishment	14.3 (2.7)	14.4 (3.2)	0.900
Burnout (Yes)	19 (43%)	16 (43%)	0.996
Work Well-Being	30.3 (7.8)	31.4 (7.3)	0.511
Work Life Balance (Dissatisfied)	16 (36%)	7 (19%)	0.127
Flourishing Scale	48.7 (5.1)	48.6 (5.4)	0.896
Satisfaction with Life Scale	25.6 (6.5)	25.5 (5.7)	0.952
Second Victim Experience (Yes)	12 (27%)	13 (35%)	0.445
Moral Injury Events Scale	22.1 (9.5)	21.9 (9.9)	0.933

Data are presented as counts (percentages) or means (standard deviation).

p-value < 0.05 was considered statistically significant.

**Table 3:** Comparison of hospitalists with and without burnout pre and during COVID-19.

		Burnout (Yes)	Burnout (No)	p-value
Emotional Exhaustion	Pre-COVID	11.8 (2.6)	6.3 (3.9)	**0.001**
	During COVID	10.1 (2.6)	7.3 (4.4)	**0.031**
Depersonalization	Pre-COVID	6.8 (5.3)	3.4 (3.0)	**0.009**
	During COVID	5.3 (3.7)	2.1 (2.8)	**0.005**
Personal Accomplishment	Pre-COVID	13.9 (2.6)	14.6 (2.9)	0.401
	During COVID	14.3 (2.7)	14.4 (3.7)	0.915
Work Well-Being	Pre-COVID	26.1 (8.8)	33.4 (5.2)	**0.002**
	During COVID	26.8 (6.7)	34.6 (5.9)	**0.001**
Work Life Balance (Dissatisfied)	Pre-COVID	11 (58%)	5 (20%)	**0.019**
	During COVID	4 (25%)	3 (14%)	0.081
Flourishing Scale	Pre-COVID	46.7 (5.9)	50.3 (3.8)	**0.019**
	During COVID	47.3 (5.2)	49.5 (5.5)	0.247
Satisfaction with Life Scale	Pre-COVID	23.7 (7.4)	27.0 (5.5)	0.095
	During COVID	22.8 (5.9)	27.7 (4.5)	**0.010**
Second Victim Experience (Yes)	Pre-COVID	10 (53%)	2 (8%)	**0.001**
	During COVID	7 (44%)	6 (29%)	0.338
Moral Injury Events Scale	Pre-COVID	24.2 (11.1)	20.5 (7.80)	0.233
	During COVID	27.3 (9.3)	17.6 (8.3)	**0.003**
Perceived COVID-Related Risk	Pre-COVID	--------------	--------------	-------------
	During COVID	7.0 (1.3)	5.8 (1.5)	**0.015**

Data are presented as counts (percentages) or means (standard deviation).

p-value < 0.05 was considered statistically significant.

Pre-COVID-19, second victim experiences were particularly prevalent in those with burnout (53%). During COVID-19, second victim experiences were still prevalent among those with burnout (44%), but the prevalence of second victim experiences was higher in those without burnout during COVID-19 (29%) than pre-COVID-19 (8%). One of the factors that strongly distinguished those with and without burnout during COVID-19 was moral injury. To further investigate this finding, an item analysis was performed to determine the items in the Moral Injury Events Scale that distinguished between those with and with burnout during the pandemic (Table 4). A variety of types of moral injury were endorsed by those with burnout during COVID-19 than those without burnout. In this context, the burned out hospitalists experienced negative impact on their moral ethics and felt betrayed by those around them that they had trusted (medical professionals, leaders and those outside the medical profession).

**Table 4:** Item analysis of the moral injury events scale during COVID-19 comparing hospitalists with and without burnout.

	Burnout (Yes) n = 15	Burnout (No) n = 20	p-value
I saw things that were morally wrong	4.1 (1.8)	2.4 (1.6)	**0.008**
I am troubled by having witnessed others’ immoral acts	4.5 (1.6)	3.2 (2.2)	0.070
I acted in ways that violated my own moral code of ethics	2.1 (1.2)	1.3 (0.9)	**0.026**
I am troubled by having acted in ways that violated my own morals or values	2.3 (1.6)	1.6 (1.4)	0.136
I violated my own morals by failing to do something that I felt should have been done	1.8 (0.9)	1.4 (0.9)	0.222
I am troubled because I violated my morals by failing to do something that I felt should have been done	1.9 (1.2)	1.6 (1.4)	0.548
I feel betrayed by leaders who I once trusted	4.3 (1.5)	2.8 (1.8)	**0.011**
I feel betrayed by fellow medical professionals who I once trusted	3.8 (1.8)	2.0 (1.7)	**0.003**
I feel betrayed by others outside of the medical profession who I once trusted	2.5 (1.6)	1.6 (1.2)	0.052

Data are presented as means (standard deviation).

p-value < 0.05 was considered statistically significant.

## Discussion

The increasing number of physicians leaving practice, especially hospitalists, has been well-documented and the most commonly examined factor associated with this exodus has been burnout [48]. By the time signs of burnout are apparent, however, it may be more difficult to effectively intervene. It is imperative that the predictors and factors associated with burnout be identified and understood, so that earlier interventions can be made. Other less studied factors that may help to identify those at risk of burnout include moral injury, well-being and prevalence of second victim experiences [25]. The COVID-19 pandemic has put a unique and unprecedented stress on hospitalists who have been at the front lines of patient care [20,29,31]. Therefore, we sought to examine burnout and other associated factors in hospitalists and subsequently how these were affected by the COVID-19 pandemic. We found that similar numbers of providers experienced burnout both pre and during COVID-19, however, the factors that were associated with burnout differed during the two time periods. Not unexpectedly, providers who reported burnout were statistically significantly more likely to experience emotional exhaustion, depersonalization and decreased work well-being regardless of presence or absence of the COVID-19 pandemic [7,12]. Each of these factors are the key dimensions in the definition of burnout [33].

Second victim experiences among hospitalist providers in the cohort demonstrated different patterns pre and during COVID-19. Specifically, while the prevalence of second victim experiences remained similar in those who experienced burnout both pre and during the pandemic, the prevalence of these experiences increased in those without burnout during the pandemic (compared to pre COVID-19). As second-victim experiences have typically been studied in extreme stress or crisis situations [18,28], we anticipated that the prevalence would increase across the board and that this would still be an important factor in distinguishing those with burnout from those without burnout during COVID-19. Though this was not seen in our sample, moral injury, which is similarly assessed in stressful scenarios, did strongly distinguish between those with and without burnout during COVID-19.

As previously mentioned, moral injury is an individual’s sense of having violated a certain moral code [27]. We hypothesize that the experience of moral injury during COVID-19 was related to a variety of factors. First, it may have been the unpredictability of workflows and constantly changing policies that affected providers’ professional and personal lives [29,31]. Providers were encouraged to limit time in hospital to minimize exposure risks. Our data illustrated that there was a significant decrease in amount of time worked per week in both clinical and non-clinical duties during COVID-19 (Table 1). Within this context, there was also the advent of telemedicine. Telemedicine certainly has benefits, but it might have also been related to moral injury, as physicians treating patients without being able to complete a full physical exam may have been objectionable. Conversations about potential for allocation of limited resources such as personal protective equipment or life-support devices were also likely sources of moral discomfort for both the lay and healthcare communities.

Item analysis of the moral injury scale provided more specific information on the particular types of moral injury that were endorsed more frequently in those with burnout during COVID-19 than pre-COVID-19. In this context, those who were burned out did experience feeling betrayed by their trusted medical professionals, as well as trusted non-medical professionals, and trusted leaders. Although the perception in this group was similar in the pre-COVID and during COVID periods, it became more related to burnout during COVID. There are a number of potential factors that could have contributed to this relationship during the COVID pandemic including policies that were being developed regarding the testing of healthcare providers, access to personal protective equipment, and the difficulty for the hospitalists to be able to care for COVID patients virtually.

Our study had some important limitations with the most notable one being that the COVID-19 pandemic has continued to be an ongoing and evolving process. Our data present a snapshot of pre-COVID-19 compared to during COVID-19. The facility where the data were collected experienced two separate COVID-19 surges. The during COVID-19 data were collected after the first surge (Spring-Summer 2020), but prior to the second surge (November 2020 to February 2021). The surges experienced by this facility were considered to be “high impact” by the U.S. Department of Health and Human Services. Though the numbers did not reach quite the same height as they did during the initial surge, the second surge was more prolonged. We may have missed providers who experienced burnout during or after the second wave, or factors associated with burnout during the second wave may have been different. Once COVID-19 vaccines were available to healthcare providers, that may have also impacted rates of burnout and moral injury. Continued examination of the longer-term consequences of COVID-19 for hospitalists is essential for long-term planning and intervention development for those at risk of burnout.

In addition, our data were collected anonymously to ensure confidentiality for the hospitalists. Thus, the matching of pre and during COVID-19 responses could not be performed. It is also possible that there were different people completing the pre versus during survey. However, there were no statistically significant characteristics (age, experience, gender, etc) that were different between the two groups, providing support for the comparability between the pre and during data collected. Additionally, the hospitalists that participated were concentrated in the metropolitan Washington, DC area and the sample size was relatively small, potentially limiting the generalizability of the findings. However, we were able to identify statistically significant differences comparing pre and during COVID-19 time points, indicating that the size of our sample was sufficient for the analyses conducted. Finally, there was no breakdown of the data with regards to how work-hours were specifically spent, particularly examining the amount of time hospitalists spent taking care of COVID-19 patients versus non-COVID-19 patients. This factor may have also impacted levels of burnout, second-victim and moral injury experiences.

This study indicates that burnout is not a “one size fits all” type of problem. While there were some factors that predicted burnout that were similar both pre- and during-pandemic, there were some that were unique to predicting burnout during COVID-19 (e.g., satisfaction with life, moral injury, and perceived COVID-19-related risk. These latter factors are particularly important as we have found that: (1) COVID-19 is already much longer lasting than initially anticipated; and (2) there will likely be future healthcare crises and other events that may put similar stress on healthcare providers. With burnout being a contributing factor to future physician shortages, it is imperative that predictive factors in a variety of different environments are well studied in order to prevent future shortages [16,17]. Hospitalists may be an excellent barometer of these factors given their presence on the front line during the pandemic. Future research will be necessary to delve into more specific factors that contribute to second victim and moral injury experiences so that targeted interventions aimed at addressing those factors may be established.
